# Is It Possible to Prepare a “Super” Anion-Exchange Membrane by a Polypyrrole-Based Modification?

**DOI:** 10.3390/membranes13010103

**Published:** 2023-01-12

**Authors:** Anton Kozmai, Mikhail Porozhnyy, Valentina Ruleva, Andrey Gorobchenko, Natalia Pismenskaya, Victor Nikonenko

**Affiliations:** Membrane Institute, Kuban State University, 149, Stavropolskaya Str., 350040 Krasnodar, Russia

**Keywords:** ion-exchange membrane, conductivity, diffusion permeability, counterion permselectivity, polypyrrole, modelling

## Abstract

In spite of wide variety of commercial ion-exchange membranes, their characteristics, in particular, electrical conductivity and counterion permselectivity, are unsatisfactory for some applications, such as electrolyte solution concentration. This study is aimed at obtaining an anion-exchange membrane (AEM) of high performance in concentrated solutions. An AEM is prepared with a polypyrrole (PPy)-based modification of a heterogeneous AEM with quaternary ammonium functional groups. Concentration dependences of the conductivity, diffusion permeability and Cl^−^ transport number in NaCl solutions are measured and simulated using a new version of the microheterogeneous model. The model describes changes in membrane swelling with increasing concentration and the effect of these changes on the transport characteristics. It is assumed that PPy occupies macro- and mesopores of the host membrane where it replaces non-selective electroneutral solution. Increasing conductivity and selectivity are explained by the presence of positively charged PPy groups. It is found that the conductivity of a freshly prepared membrane reaches 20 mS/cm and the chloride transport number > 0.99 in 4 M NaCl. A choice of input parameters allows quantitative agreement between the experimental and simulation results. However, PPy has shown itself to be an unstable material. This article discusses what parameters a membrane can have to show such exceptional characteristics.

## 1. Introduction

The main functional property of ion-exchange membranes (IEMs) is their selectivity with respect to the transport of counterions. The efficiency of desalination and concentration of electrolyte solutions by electrodialysis methods mainly depends on this property [1,2,3].

Due to the growing demand for the treatment of wastewater, recovery and concentration of valuable ion components [4,5,6], production of fertilizers [5,7], energy production by reverse electrodialysis [8,9] fuel cells [10,11,12], flow batteries [12,13,14] and other processes that involve IEM [15,16,17,18], the need for high-selective IEMs is growing rapidly.

In the literature, there are a number of studies aimed at increasing the selectivity of IEMs in relation to the counterion transport both by synthesizing new membranes [19,20] and by modifying commercially available ones [21,22,23,24]. Attempts to increase selectivity often lead to a decrease in IEM conductivity [25]. Conversely, modification or synthesis of membranes that results in an increase in conductivity is accompanied by a decrease in selectivity. Generally, an increase in conductivity can be achieved by increasing the membrane water content, which negatively affects its selectivity. To ensure high selectivity, the pore size must be small so that their volume is completely occupied by an electric double layer, in which there are practically no coions. As for the conductivity, its high values, especially at high external solution concentrations, on the contrary, are due to the presence of large pores containing an electrically neutral solution. This problem in the literature is called “the trade-off between membrane selectivity and conductivity” [5,25,26]. It concerns not only counterion permselectivity, but permselectivity between two different species, which can be different gas molecules [27] or competing counterions [25,26].

It is possible to simultaneously increase the IEM selectivity and conductivity by increasing their exchange capacity; however, this method can lead to a decrease in the membrane mechanical strength due to an increase in its osmotic pressure and a tendency to break polymer chains. The solution to the problem can be an increase in the exchange capacity in combination with an increase in the degree of cross-linking of the polymer [25], which will limit the increase in the degree of membrane swelling. Another strategy to overcome the trade-off is to obtain hybrid membranes by introducing inorganic nanoparticles into the IEM structure. In some cases, this approach makes it possible to simultaneously increase the conductivity and reduce the water content [28], as well as increase the selectivity [23].

Despite the fact that the technologies of IEM production are constantly being improved, the development of new types of membranes is quite expensive. As a result, their number remains limited [5]. A promising direction for obtaining new membranes with high performance is the modification of commercial membranes, which is carried out using a small amount of modifying agents and, as a rule, leads to only a slight increase in the membrane cost. There are many studies that use this approach to the development of monovalent-ion-selective membranes [15,25,29,30,31,32,33]. However, only a few studies where this approach is used are devoted to improving the membrane permselectivity with respect to counterions [34,35,36].

In order to improve the membrane properties, the idea of clogging the membrane’s non-selective macropores with a homogeneous microporous ion-exchange material was realized in some studies [24,35,37,38]. This idea is based on the fact that relatively large (macro)pores are the pathway for coions through the membrane [35,39,40]. The realization of this approach leaded to a 2.5-time decrease in the coion transport number in an anion-exchange membrane (CJMA-7, Hefei Chemjoy Polymer Materials Co., Ltd., Hefei, China) after its modification with the sulfonated fluoropolymer MF-4SK (JSC “Plastpolymer”, Saint Petersburg, Russia) [35]. The polypyrrole (PPy)-based modification of an FAA-3-50 membrane (Fumatech BWT Company, Bietigheim-Bissingen, Germany) resulted in a twofold increase in membrane conductivity [38].

The literature shows that PPy-based modification is mainly used to obtain monovalent-ion-selective membranes [41,42]. As far as we know, only the paper of Salmeron-Sanchez et al. [37,38] on PPy-based modification of IEMs was aimed at improving other membrane properties, namely, membrane conductivity, and this task was successfully solved in relatively dilute NaCl solutions (up to 0.04 M). Note that the authors used the microheterogeneous model (MHM) [43] to interpret their results. This model suggests that IEM can be considered as a two-phase system consisting of a microporous gel phase and a solution that fills the intergel spaces. The change in membrane properties after modification with PPy is explained by the presence of anion-exchange fixed groups in PPy, which leads to an increase in the total exchange capacity. From the point of view of the model, the presence of PPy causes an increase in the volume fraction of the gel phase and a decrease in the content of the electrically neutral solution filling the intergel spaces [38], which should lead to an increase in counterion permselectivity.

In this paper, we compare the properties of the heterogeneous anion-exchange membrane MA-41 with its PPy-based modifications MA-41_mod_ and MA-41_modED_. The MA-41_mod_ membrane is a membrane freshly obtained after modification; the MA-41_modED_ membrane is an MA-41_mod_ one used in an ED process. Like Salmeron-Sanchez et al. [38], we apply a modification of the MHM [43] to describe the properties of the membranes under study. In addition to the membrane conductivity studied in Ref. [38], we examined the membrane diffusion permeability, counterion permselectivity (the Cl^−^ transport number) and thickness as functions of the bathing solution concentration. We developed a new version of the MHM, which takes into account the presence of PPy in macro- and mesopores via its equivalent volume in dry form and its exchange capacity. This MHM version also accounts for the loss of water by the membrane with an increase in the bathing solution concentration and associated decrease in the swelling degree. The model allows us to obtain a quantitative agreement between the simulation and experiment in a wide concentration range from 0.1 M to 4 M NaCl.

## 2. Materials and Methods

### 2.1. Experiment

#### 2.1.1. Pristine Membrane

The commercial heterogeneous anion-exchange membrane MA-41 (JSC “Shchekinoazot”, Pervomayskiy, Russia) is used for preparing polypyrrole-modified samples. The MA-41 membrane is produced by hot pressing of a mixture of polyethylene (inert binder) and powdered AV-17 ion-exchange resin (styrene-divinylbenzene copolymer) (LLC “Ural Chemical Company”, Chelyabinsk, Russia) with nylon reinforcing cloth. Ion-exchange groups are represented mainly by quaternary amines with a small amount of tertiary and secondary amines [44]. The membrane contains polyethylene as a binder and reinforcing net of polyamide filaments.

#### 2.1.2. Membrane Modification

To obtain the modified samples of MA-41 with homogeneously existing polypyrrole (PPy) across the membrane thickness, the following protocol was used. First, membranes were immersed in an aqueous 0.4 mol/L solution of pyrrole (Py) (LLC “Merck”, Moscow, Russia) for 24 h to let the samples soak and equilibrate with the solution. Then, without any blotting, the samples were immersed in an aqueous 0.5 mol/L FeCl_3_ (JSC “LenReaktiv”, Saint Petersburg, Russia) solution for 24 h. The latter allowed chemical polymerization of Py in situ in the membrane’s porous medium using FeCl_3_ as the oxidizing agent.

**Figure 1:**
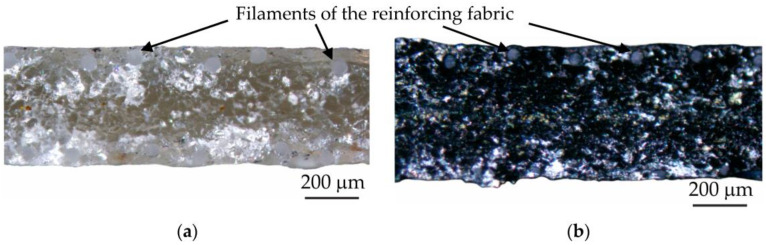
Optical images of the cross-section of the (**a**) pristine MA-41 membrane and (**b**) PPy-modified MA-41_mod_ membrane in the swollen state.

In this paper, three membranes were characterized: the pristine MA-41 membrane, the freshly PPy-modified membrane (MA-41_mod_) and a MA-41_mod_ membrane that was used in ED (MA-41_modED_) at current densities close to or higher than the limiting current density during 5 h. Measurement of the current–voltage curves of the MA-41_modED_ membrane showed that these curves did not change after 5 h of ED. Therefore, it can be assumed that a five-hour membrane operation under conditions of intensive current ED is sufficient to stabilize the electrochemical properties of this membrane. The optical images of the cross-section of the MA-41 and MA-41_mod_ membranes are shown in Figure 1.

#### 2.1.3. Membrane Characterization

Membrane conductivity and diffusion permeability were measured at 25 °C.

##### Electrical Conductivity

The values of electrical conductivity for the membranes studied were determined from the resistance measured using a differential method with the laboratory clip cell [45] and the MT4080A immittance meter (Motech Industries Inc., New Taipei, Taiwan) at the alternating current frequency of 1 kHz.

##### Diffusion Permeability

The diffusion permeability coefficients were determined using a laboratory two-compartment flow cell [46]. A NaCl solution of a known concentration and neutral pH was pumped through one of the compartments, and deionized water was pumped through the other. Determination of the electrolyte flux, J, across the membrane separating two compartments allowed calculation of its integral diffusion permeability coefficient:

(1) P=Jd/c,

where *c* is the electrolyte solution concentration (NaCl) *d* is the membrane thickness.

Knowing P, it is possible to calculate the differential diffusion permeability coefficient, P*, and ion transport numbers, $t_{+}^{*}$ and $t_{-}^{*}$, in the membrane as follows [35,40]:

(2) $$P^{*}=P+c\frac{dP}{dc},$$

and

(3) $$t_{-}^{*}=\frac{1}{2}+\sqrt{\frac{1}{4}-\frac{P_{}^{*}F^{2}c}{2RT\kappa^{*}}},\;t_{+}^{*}=1-t_{-}^{*}$$

where the subscripts + and − refer to cation and anion, respectively.

##### Water Content

To determine the water content, the membrane samples preliminarily equilibrated with deionized water were gently blotted and placed in the MB25 moisture-content analyzer (Ohaus, Parsippany, NJ, USA), which was used to measure the weight of the samples in a wet (*m_w_*) and dry (*m_d_*) state. To bring the sample to a dry state, it was subjected to a temperature of 80 °C until the weight of the sample ceased to change. The water content (WC, %) was calculated as follows: $WC=\left(m_{w}-m_{d}\right)/m_{w}$.

##### Ion-Exchange Capacity

First, a sample of known weight was converted into the Cl^−^ form and equilibrated with deionized water. Second, this sample was immersed in 250 mL of a 1 M KNO_3_ solution for 24 h under constant stirring. After that, the amount of substituted Cl^−^ ions was determined with the ion chromatography system DIONEX ICS-3000 (Dionex Corporation, Sunnyvale, CA, USA).

#### 2.1.4. Polypyrrole Properties

**Figure 2:**
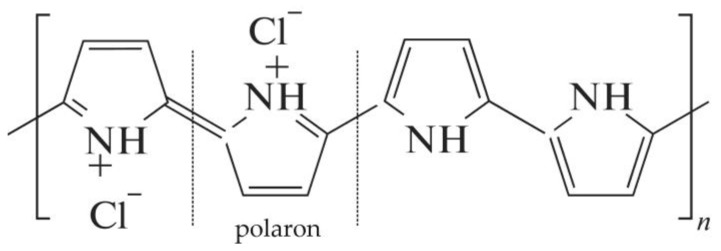
PPy structure.

PPy is an electrically conductive polymer; it has an organic structure and at the same time has properties inherent in semiconductors: redox activity and electronic and ionic conductivity. Polypyrrole is a hydrophobic material, although it exhibits anion exchange properties. The conductive properties of polypyrrole are provided by protonated oxidized units (Figure 2) [47]. Figure 2 shows the ion-conductive oxidized form of PPy, where the charged groups are positive polarons stabilized by chlorine anions.

PPy can undergo protonation/deprotonation, causing a change in its oxidation form and, as a result, the electrical conductivity can vary over a wide range (from 10^−6^ to 10 S/cm) [48]. Its value is also greatly influenced by the method by which the PPy was obtained (using various surfactants, dyes, etc.) [47,48], as well as by its morphology (nanotubes, globules, etc.) [49].

As for PPy’s ion-exchange capacity, the maximum theoretical value estimated in [50] was 3.4 mmol g^−1^. Taking into account PPy’s density of 1.5 g/cm^3^ dry PPy [51], this value is equal to 5.1 mmol/cm^3^ dry PPy. It is known that PPy undergoes deprotonation as a result of attack by OH^−^ ions. The deprotonation leads to a decrease in the concentration of the positively charged fixed groups [52] and opening of the pentacyclic rings in the PPy chains [53,54].

### 2.2. Theoretical Modelling

The developed model is a modification of the model published earlier [55,56] The modified model takes into account the change in the membrane’s degree of swelling. Below we present the main elements of this model.

The studied commercial heterogeneous anion-exchange membrane MA-41 contains mainly quaternary ammonium groups [44,56]. These groups are considered to be permanently positively charged regardless of the pH value. In addition, the membrane contains up to 20% of primary, secondary and tertiary amines. In conditions of membrane characterization (measurement of conductivity and diffusion permeability, Section 2.1.3), the solution pH does not change; hence, we consider the total ion-exchange capacity due to all functional groups.

#### 2.2.1. Model Description

##### Membrane Swelling

The system under study consists of an IEM in equilibrium with a binary electrolyte (NaCl) solution of a given concentration at neutral pH. The model considers a microporous gel (assumed to be homogeneous), as well as meso- and macropores, as structural elements of the membrane. The latter may include structural defects and voids.

The membrane swelling is due to the presence of bound water in micro- and mesopores. We will assume that bound water forms the hydration shells of fixed hydrophilic groups of the membrane and mobile ions. The amount of bound water in macropores is negligible, so these pores do not contribute to swelling.

According to Gregor’s model [57], the osmotic pressure is due to the presence of bound water; its value is greater the greater the fraction of bound water in the micro- and mesopores of the ion-exchange material:

(4) $$\pi_{IX}=\frac{RT}{{\bar{V}}_{w}}\ln\frac{q_{w}+q_{wb}}{q_{w}},$$

where ${\bar{V}}_{w}$ is the molar volume of water; $q_{w}$ and $q_{wb}$ are the number of moles of free and bound water in one equivalent of the polymer gel matrix, respectively; and *R* and *T* are the gas constant and temperature, respectively.

Let the equivalent volume of dry gel be $V_{Rdry}$ (the volume that contains 1 equiv. of fixed groups with counterions, and associated polymer chains [1]):

(5) $$V_{Rdry}=V_{R0}+V_{Cl},$$

where $V_{R0}$ (in cm^3^/mmol dry gel) is the equivalent volume of dry gel without counterions (in the case of an anion-exchange membrane, these are Cl^−^ ions); $V_{Cl}$ is the crystallographic volume of a Cl^−^ ion.

We consider the dry gel as part of a dry membrane. The dry gel is a dense material not containing pores. The dry membrane contains the dry gel and macropores, which are defects of the structure, voids, etc. When the membrane is in an electrolyte solution, it absorbs water. Water moves first towards fixed ions and counterions; this leads to the formation of micro- and mesopores. The expansion of micro- and mesopores allows “free” water to penetrate into the membrane. The number of moles of bound water per 1 equiv. of fixed ions in the swollen gel can be calculated with the equation

(6) $$q_{wb}=\left(h_{R}\bar{Q}+h_{Cl}{\bar{c}}_{Cl}+h_{Na}{\bar{c}}_{Na}\right)/\bar{Q},$$

if we know the values of the hydration numbers of the membrane fixed groups, $h_{R}$, and single charged ions in the membrane, $h_{i}$, (*i* = Cl^−^, Na^+^), as well as the gel exchange capacity, $\bar{Q}$, and the concentration of ion *i* in the membrane gel phase ${\bar{c}}_{i}$ (*i* = Cl^−^, Na^+^) (in mol/L swollen gel).

The swollen gel contains micro- and mesopores. Its volume comprising 1 equiv. of fixed ions is

(7) $$V_{Rwet}=V_{Rdry}+\left(q_{w}+q_{wb}\right){\bar{V}}_{w},$$

The amount of free water in this volume, $q_{w}$, depends on the mechanical balance of the swollen membrane: the osmotic pressure exerted by the bound water (Equation (6)) is balanced by the external solution osmotic pressure, $\pi_{sol}$, and by the restoration force, $F_{rest}$, which resists the matrix stretching:

(8) $$\pi_{mb}=\pi_{sol}+F_{rest},$$

According to van’t Hoff’s law

(9) $$\pi_{sol}=2cRT.$$

Hooke’s law assumes that $F_{rest}$ is proportional to the relative elongation ε: $F_{rest}=\varepsilon E$, where *E* is Young’s modulus characterizing the resistance of a polymer matrix to tensile stress. The difference between the osmotic pressures in the membrane and in the solution can be expressed as $\Delta \pi =\pi_{mb}-\pi_{sol}=\pi_{IX}(1-f_{macro})-\pi_{sol}$; then

(10) ε=Δπ/E,

where the $\left(1-f_{macro}\right)$ factor takes into account the fact that the macropores do not contribute to the osmotic pressure of the membrane. As a result, the expression for $V_{Rwet}$ takes the form [56]:

(11) $$V_{Rwet}=V_{Rdry}{\left(1+\varepsilon \right)}^{3}.$$

To calculate the number of moles of bound water in the membrane gel (Equation (6)), it is necessary to know the membrane exchange capacity (concentration of fixed groups in the membrane) and the concentration of mobile ions in this gel. According to the basic version of the MHM, the gel phase of the membrane is in thermodynamic equilibrium with an electrically neutral solution that fills the central parts of the macro- and mesopores. This equilibrium is expressed by the Donnan relation:

(12) $${\bar{c}}_{Cl}{\bar{c}}_{Na}=K_{D}c_{Cl}c_{Na}=K_{D}c^{2},$$

where $K_{D}$ is the Donnan constant, the overbar refers to the gel phase and the absence of the overbar refers to the electroneutral solution. In the pore electroneutral solution $c_{Cl}=c_{Na}=c$, where *c* is the external solution concentration in equilibrium with the membrane.

Equation (12) is supplemented by the electroneutrality condition in the gel phase:

(13) $${\bar{c}}_{Cl}=\bar{Q}+{\bar{c}}_{Na},$$

where $\bar{Q}=Q/f_{1}$ is the concentration of fixed ions per unit volume of the gel phase and $f_{1}$ is the volume fraction of the microporous gel phase in the membrane not containing electroneutral solution in the central parts of the mesopores.

Substitution of Equations (4)–(7), (9), (10), (12) and (13) in Equation (11) gives an algebraic expression for $q_{w}$, whose solution with known parameters $V_{Rdry}$, Q, E, $K_{D}$, $h_{R}$, $h_{i}$ (*i* = Cl^−^, Na^+^) and $f_{1}$ makes it possible to calculate the values of $q_{w}$ and $V_{Rwet}$ for a given external electrolyte solution concentration *c*. One can also calculate the total amount of water in the equivalent volume of the membrane and its water content [55].

##### Calculation of Pristine Membrane Transport Characteristics

The main transport equations of the basic microheterogeneous model are presented in a number of papers, for example [38,43,56]. Using the irreversible thermodynamics and effective medium approach, the following equations are derived [43] under the assumption that only two ions (e.g., Na^+^ and Cl^−^) are present in the solution and in the membrane:

(14) $$L_{i}=D_{i}c_{i}/\left(RT\right),\;{\bar{L}}_{i}={\bar{D}}_{i}{\bar{c}}_{i}/\left(RT\right),$$

(15) Li∗=(f1L¯iα+f2Liα)1α,

(16) $$\kappa^{*}=F^{2}\left(L_{Na}^{*}+L_{Cl}^{*}\right)$$

(17) $$t_{i}^{*}=L_{i}^{*}/\left(L_{Na}^{*}+L_{Cl}^{*}\right),$$

(18) $$P^{*}=\frac{2RTt_{Cl}^{*}L_{Na}^{*}}{c}=\frac{2RT\kappa^{*}t_{Na}^{*}t_{Cl}^{*}}{F^{2}c},$$

where $L_{i}$, $D_{i}$ and $c_{i}$ are the transport coefficient, diffusion coefficient and concentration of ion *i* (*i* = Cl^−^, Na^+^), respectively; $f_{1}$ and $f_{2}$ are the volume fractions of the gel phase and intergel spaces filled with the electroneutral solution in the membrane ($f_{1}+f_{2}=1$), respectively; $c=z_{+}c_{+}=-z_{-}c_{+}$ is the electrolyte concentration in the external equilibrium solution; $t_{i}^{*}$ is the ion transport number in membrane; $\kappa^{*}$ is the conductivity; $P^{*}$ is the local coefficient of diffusion permeability; α is the structural parameter, which reflects the arrangement of the phases constituting the membrane (two limiting cases are possible: α = 1 corresponds to the parallel arrangement of phases relative to the transport axis; α = −1 corresponds to the series arrangement); the asterisk and the overbar refer to the membrane as a whole and to the gel phase, respectively; the absence of overbar refers to the electroneutral solution; and *R*, *T* and *F* are the gas constant, absolute temperature and Faraday constant, respectively.

In Equations (14) and (15) ${\bar{D}}_{i}$, $f_{2}$ and α are considered to be dependent on the degree of membrane swelling as described below.

**Figure 3:**
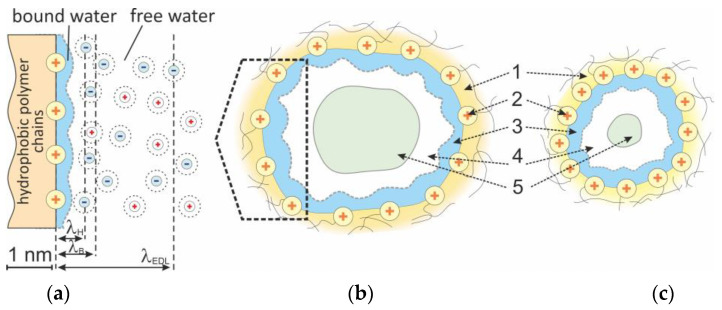
The schematic representation of micro- or mesopore structure showing the cases of a low (**c**) and a high (**b**) degree of swelling. The numbers denote hydrophobic polymer chains (1), fixed ions (2), bound water (3), free water (4) and electroneutral solution (5). In figure (**a**), λ_H_ is the Helmholtz length, λ_B_ is the Bjerrum length and λ_EDL_ is the length of the electric double layer.

With an increase in the external solution concentration, its osmotic pressure increases. That is, the force opposing the membrane swelling grows. The membrane swelling degree decreases: part of the free water leaves the membrane; as a result, $\pi_{mb}$ increases and a new equilibrium is reached. A decrease in the degree of swelling is expressed in a decrease in the pore size, which leads to a decrease in the parameter $f_{2}$. To account for this effect, we represent $f_{2}$ as

(19) $$f_{2}=f_{macro}+f_{2meso},$$

where $f_{2meso}$ is the volume fraction of the electroneutral solution localized in the membrane mesopores (it occupies the central part of the pores, outside the EDL, Figure 3, Redrawn from [56]).

Since macropores contain almost no bound water and do not contribute to the membrane osmotic pressure, their volume is assumed to vary proportionally to the volume of the membrane matrix, i.e., $f_{macro}$ does not depend on *c*. With an increase in *c*, the volume of the electroneutral solution in the mesopore decreases, because mesopore radius decreases (the mesopore loses free water). However, the thickness of the electrical double layer nearly does not change, so that the fraction of the charged solution in the mesopore increases; accordingly, the volume fraction of the electroneutral solution inside the mesopore decreases. To account for this effect, we will use the empirical formula:

(20) $$f_{2meso}=f_{w}\eta ,$$

where

(21) $$\eta =1-e^{1/\left(f_{w}-1\right)}$$

and

(22) $$f_{w}=V_{w}q_{w}/V_{tot}.$$

In Equations (20)–(22), $f_{w}$ is the ratio of the total free water (in micro- and mesopores) volume to the membrane volume. The membrane volume is calculated as

(23) $$V_{tot}=V_{Rwet}/\left(1-f_{macro}\right).$$

**Figure 4:**
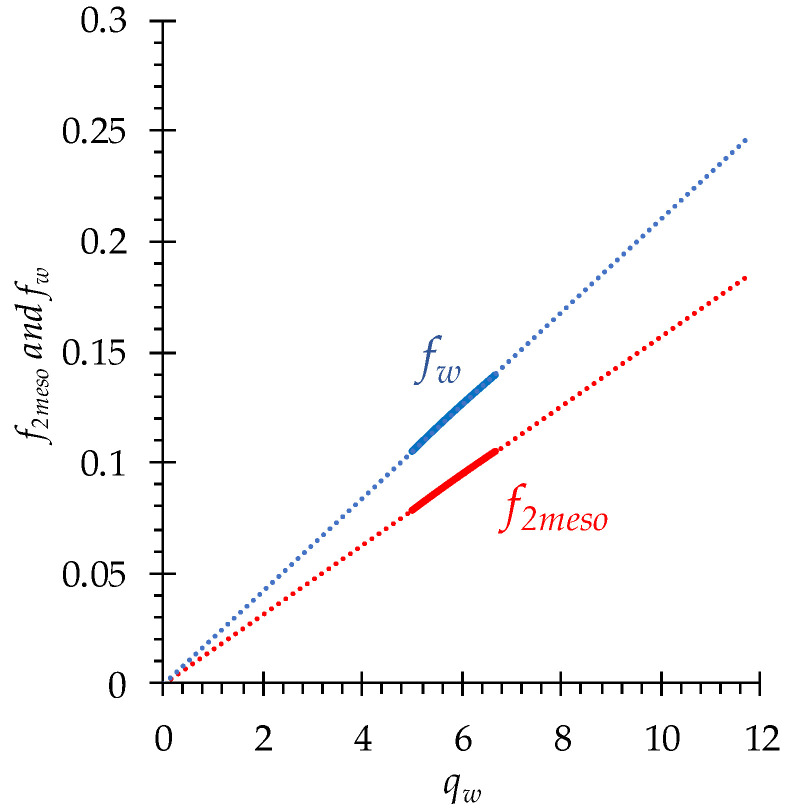
*f*_2*meso*_ and *f_w_* as functions of the amount of free water, *q_w_*, in the MA-41 membrane. The range of *q_w_* and calculated *f*_2meso_ and *f*_w_ values corresponding to the solution concentrations from 0.1 to 4 mol/L are shown by solid lines. Extrapolation of simulation data is shown by dots.

If we imagine that the micropores of the gel phase do not contain free water, and all free water is in the mesopores (macropores are not considered), then the $f_{2meso}$ value will be only slightly less than the value of $f_{w}$, because the volume of the electroneutral solution in the mesopore is slightly less than the volume of free water (Figure 4). Bound water makes up the hydration shells of fixed ions, i.e., it enters the electrical double layer, although free water is present there at a distance of approximately 0.5 nm from the charged wall [58] (Figure 3). The factor η in Equation (20) takes into account the fact that with a decrease in the free water fraction, the difference between $f_{meso}$ and $f_{w}$ decreases.

With a decrease in the pore size, the ion mobility decreases due to an increase in the constraint of ion channels and an increase in the tortuosity of the ion pathways. According to Mackie and Meares [59], the effective value of the ion diffusion coefficient, ${\bar{D}}_{i}$, in the gel phase is determined by the expression

(24) $${\bar{D}}_{i}={\bar{D}}_{i}^{\prime }\zeta /\bar{\zeta },$$

(25) $$\zeta ={\left(1-f_{m}\right)}^{2}/{\left(1+f_{m}\right)}^{2},$$

(26) $$f_{m}=V_{Rdry}/V_{Rwet}$$

where ${\bar{D}}_{i}^{\prime }$ (a fitting parameter) is a character value of ${\bar{D}}_{i}$ (achieved at *c* = 1 M), ζ is the tortuosity coefficient, $\bar{\zeta }$ is the value of ζ at *c* = 1 M, and $f_{m}$ is the volume fraction of the swollen polymer matrix impermeable for diffusion.

Mackie and Meares assume that ${\bar{D}}_{i}^{\prime }$ takes the same value as an ion diffusion coefficient in free solution ($D_{i}^{0}$), which is acceptable for relatively big pores. In our model, ${\bar{D}}_{i}^{\prime }<D_{i}^{0}$ to account for the tightness of ion-conducting pores; ${\bar{D}}_{i}^{\prime }$ is independent of the membrane swelling degree.

In addition to a decrease in the ion diffusion coefficients, an increase in the tortuosity of the ion path leads to an increase in the number of series gel/electroneutral solution arrangements, i.e., to a decrease in the value of the parameter α. To take this effect into account, we use an empirical equation similar to Equation (24):

(27) $$\alpha =\alpha^{\prime }\zeta /\bar{\zeta }.$$

where $\alpha^{\prime }$ is the fitting parameter, and ζ and $\bar{\zeta }$ have the same meaning as above.

Thus, the algorithm for calculation of the IEM transport characteristics, taking into account the change in the membrane matrix structure with an increase in the bathing solution concentration, is as follows. For each given concentration of the bathing solution, first the set of equations describing the membrane swelling is solved [Equations (4)–(7), (9)–(13)]. This allows one to determine the number of moles of free and bound water in the membrane matrix, as well as its equivalent volume. Knowing these parameters makes it possible to further calculate the volume fraction of mesopores, $f_{2meso}$ [Equations (20)–(22)], as well as ${\bar{D}}_{i}$ (*i* = Cl^−^, Na^+^) [Equations (24)–(26)] and α [Equation (27)]. Finally, Equations (14)–(18) allow calculation of the conductivity, transport number and diffusion permeability. The determination of the model input parameters is discussed in Section 3.1.

##### Accounting for the Presence of PPy

**Figure 5:**
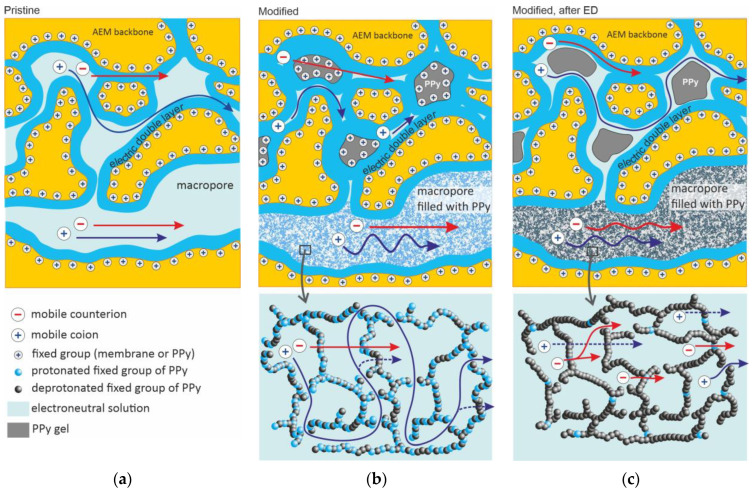
Schematic representation of the changes in studied membrane structure at different stages of its treatment: (**a**) the pristine MA-41 membrane, (**b**) the modified membrane, (**c**) the modified membrane used in an ED process. The membrane structure is presented in accordance with modern concepts [58,60,61]: mesopores (ion clusters) are connected to each other by narrower ion-conducting channels; the macropores are due to structural defects including the gaps between ion-exchange material and non-conductive fillers, such as fibers of reinforcing cloth [40]. The arrows show ion pathways during salt diffusion through the membrane.

PPy is a polymer whose chains carry fixed positively charged ions. An increase in the conductivity and a decrease in the diffusion permeability of the PPy-modified membrane are explained by the fact that PPy penetrates into macro- and mesopores and replaces the electrically neutral solution there (which reduces the membrane permselectivity). The PPy polymer chains form a kind of selective wall that separates relatively large pores into smaller compartments (Figure 5). In this case, in order for ions to cross the pore space, it is necessary to cross the selective walls. These walls can be easily passed by anions, but they serve as barriers for cations.

A previously developed model approach [62] is used in accounting for the presence of PPy in the membrane. In this approach the modified membrane structure is represented as an “ion exchanger inside ion exchanger”. To calculate the effective conductivity coefficient of the membrane, $L_{i}^{*}$, Equation (15) is used. However, in this equation, $L_{i}$ now is not the coefficient characterizing the electrically neutral solution in the pore, but the conductance coefficient of the effective medium, which is a swollen ion exchanger—PPy. It is assumed that PPy occupies the volume that was previously occupied by an electrically neutral solution in a non-modified membrane: the macropores and central parts of mesopores with a total volume fraction *f*_2_. Similar to the entire membrane in the non-modified form, the gel phase and the intergel spaces filled with an electrically neutral solution are considered to exist in the swollen PPy (Figure 5). To calculate the ion transport coefficients in PPy, we will also use a system of equations of the form of (14)–(18):

(28) $${\bar{L}}_{i}^{PPy}={\bar{D}}_{i}^{PPy}{\bar{c}}_{i}^{PPy}/\left(RT\right),$$

(29) Li∗PPy=(f1PPy(L¯iPPy)αPPy+f2PPyLiαPPy)1αPPy.

As before, the overbar refers to the gel phase (of the PPy in this case); quantities without an overbar refer to the phase of an electrically neutral solution present in relatively large pores of PPy.

Knowing $L_{i}^{*PPy}$, it is possible to calculate the ion transport coefficients in the modified membrane (index “mod”):

(30) Li∗mod=(f1mod(L¯imod)αmod+f2mod(Li∗PPy)αmod)1αmod.

After calculation of $L_{i}^{*\mathrm{mod}}$ using Equation (30), the values of $\kappa_{i}^{*\mathrm{mod}}$, $t_{i}^{*\mathrm{mod}}$ and $P_{i}^{*\mathrm{mod}}$ are calculated with Equations (16)–(18).

Both the swelling of PPy and the swelling of the host membrane matrices result in stretching of the system of polymer chains of these materials. The model assumes that when PPy swells inside the host membrane, it exerts additional pressure, which tends to stretch the host membrane matrix. Thus, Equation (8), which expresses the equality to zero of all acting forces on the host membrane matrix, and Hooke’s Equation (10) are still used for the calculation of $q_{w}$. The difference is that $\pi_{mb}$ is understood as the sum of the forces applied to the host membrane matrix, exerted by both the host membrane matrix and the nested PPy. In this case, the difference in osmotic pressures exerted on the membrane matrix by bound water inside and outside the membrane will be equal to:

(31) $$\Delta \pi =\pi_{IX}(1-f_{2}^{MA-41}f_{macro}^{PPy})-\pi_{sol}$$

where $\pi_{IX}$ is the osmotic pressure in an ion exchanger which is understood here as the gel phase formed by both fixed groups of the host membrane and guest PPy. As before, it is believed that only micro- and mesopores can exert osmotic pressure. PPy occupies a volume fraction $f_{2}^{MA-41}$ in the host membrane; it contains macropores with a volume fraction $f_{macro}^{PPy}$ (relative to the volume of PPy itself).

In the first stage of calculations, we find the parameters of membrane swelling. Equation (4) is used to calculate $\pi_{IX}$. In this equation $q_{w}$ and $q_{wb}$ are the number of moles of free and bound water in one equivalent of the MA-41 gel matrix including the contributions caused by the presence of fixed ions of both MA-41 and PPy. The $q_{wb}$ value is found as follows:

(32) $$q_{wb}^{\mathrm{mod}}=\left(\begin{array}{l} \left(h_{R}^{MA-41}{\bar{Q}}^{MA-41}+h_{Cl}{\bar{c}}_{Cl}^{MA-41}+h_{Na}{\bar{c}}_{Na}\right)(1-f_{2}^{MA-41})+\\ \left(h_{R}^{Ppy}{\bar{Q}}^{PPy}+h_{Cl}{\bar{c}}_{Cl}^{PPy}+h_{Na}{\bar{c}}_{Na}^{PPy}\right)f_{2}^{MA-41}(1-f_{2}^{PPy}) \end{array}\right)/Q_{}^{MA-41},$$

The volume of the swollen modified membrane is then

(33) $$V_{Rwet}^{\mathrm{mod}}=V_{Rdry}^{\mathrm{mod}}+V_{Rdry}^{PPy}+\left(q_{w}+q_{wb}\right){\bar{V}}_{w},$$

where, compared to Equation (7), we take into account the volume of dry PPy, $V_{Rdry}^{PPy}$, nested in the membrane.

When using Equation (10) with the value of Δπ determined with Equation (31), and replacing Equation (6) with Equation (32) and Equation (7) with Equation (33), we obtain a similar algebraic equation system to find *q_w_*. By solving this equation system, we also find the equivalent volume of the modified membrane at a given concentration and the relative elongation of the membrane, *ε*, due to its swelling.

In the second stage, we calculate the transport characteristics of the modified membrane. To apply Equations (14)–(18) and (28)–(30) for this purpose, it is necessary to know not only the structural and kinetic parameters of the host membrane, but also the similar parameters of the nested PPy. As in the case of the pristine membrane, we take into account that with increasing concentration of the bathing solution, the host membrane and nested PPy lose a part of the free water. This causes narrowing of the pores (and a decrease in parameters $f_{2meso}$ and $f_{2}$) as well as a decrease in the ionic diffusion coefficients in the gel phases of the host membrane and PPy. To do this, we calculate the values of parameters $f_{2meso}$ and $f_{2}$, as well as the tortuosity coefficient, ζ, using the same Equations (19)–(23), (24) and (25) through the values of $q_{w}$ and $q_{wb}$ found in the previous calculation step. The difference is in the application of Equation (26) for calculating the volume fraction of the hydrophobic polymer chains, *f_m_*. In the case of the host membrane, we use Equation (26) without changes. However, for calculation of the similar parameter for PPy, Equation (26) is replaced with the following equation

(34) $$f_{m}^{PPy}=\frac{V_{Rdry}^{PPy}}{V_{Rwet}^{PPy}}=\frac{V_{Rdry}^{PPy}}{V_{tot}^{\mathrm{mod}}f_{2}^{\mathrm{mod}}}$$

Note that to have the same value of $f_{2}$ at a given concentration *c* in the first and second calculation stages, we perform several iterations: we take a tentative value of *f*_2_ to calculate the parameters of the swollen membrane, then apply Equations (19)–(23) to find the reduction in *f*_2_ due to the increase in the concentration, then use the new value of *f*_2_ to repeat the loop until *f*_2_ stops changing.

## 3. Results

### 3.1. Determination of the Input Parameters

The model input parameters involve thermodynamic, structural, kinetic and mechanical parameters characterizing (1) the pristine MA-41 membrane, (2) the host membrane right after modification (MA-41_mod_), (3) the host membrane used in an ED process (MA-41_modED_); (4) the freshly synthesized PPy and (5) the PPy after the use of the modified membrane in an ED process (PPy_ED_). The PPy modification leads to an increase in the membrane thickness, i.e., to a stretch of the MA-41 membrane matrix. As mentioned in Section 2.1.4, PPy is deprotonated by attack of OH^−^ ions. During ED under intensive current regimes, these ions are generated in the water-splitting reaction [63,64,65] occurring in the membrane/depleted solution interface. The H^+^ ions formed in this reaction are released into the depleted solution, and OH^−^ ions move through the AEM towards the concentrate compartment. The presence of OH^−^ ions in the PPy-modified membrane leads to a decrease in the concentration of the positively charged fixed groups [52] and opening of the rings of the PPy chains [53,54]. The decrease in the ion-exchange capacity of the PPy after the use of the membrane in ED leads to a decrease in its swelling, which causes a decrease in the thickness of the MA-41_modED_ membrane.

Table 1 shows all the input parameters. Some of them were found experimentally or evaluated theoretically. The ion-exchange capacity of the pristine MA-41 membrane, *Q*, (equal to the concentration of fixed ions per unit volume of the membrane) was determined as described in Section 2.1.3 and equal to 1.47 mmol/cm^3^ wet membrane. The determined value is close to values reported in Refs. [40,55]. It is assumed that the *Q* value for the host membrane matrix remains unchanged even in the presence of PPy in the membrane pores. The maximum possible exchange capacity of PPy, theoretically determined in Ref. [50], is equal to 5.1 mmol/cm^3^ dry PPy. As our simulation shows (Section 3.2), the total amount of water in PPy is about 10 mol H_2_O/mol fixed ions. Therefore, 1 cm^3^ of dry PPy should contain about 50 mmol or slightly less than 1 cm^3^ of H_2_O. In fact, the volume of water held by one functional group of PPy should be essentially lower than the volume of water held by one functional group of the host membrane matrix, due to the more hydrophobic nature of PPy [52] as compared to the IEM matrix. Thus, the concentration of fixed ions in wet PPy not containing macropores should be more than 2.5 mmol/cm^3^ wet PPy. For calculations, we take 2 mmol/cm^3^ wet PPy (not containing macropores) for the freshly prepared PPy inside the MA-41_mod_ membrane and 0.05 mmol/cm^3^ wet PPy inside the MA-41_modED_ membrane.

The evaluation of $V_{Rdry}$ for the host matrices of the studied membranes and for PPy is given in the Supplementary Materials.

The characteristic values of the parameter describing Donnan equilibrium, *K_D_*, can be estimated when considering its dependence on the water content in the membrane as described in [55]. When writing Equation (12) for the concentrations expressed in mmol per cm^3^ of free water within the membrane pores (${\bar{c}}_{i}^{mol}$), the Donnan constant should be on the order of 1. For this, we assume that the bound water in the pore solution does not take part in the equilibrium relations [1], and there are no specific interactions of ions with the membrane polymer matrix. In our model, we use the ion concentrations expressed in mmol per cm^3^ of swollen membrane, ${\bar{c}}_{i}$. The latter is linked to ${\bar{c}}_{i}^{mol}$ by the relation

(35) $${\bar{c}}_{i}={\bar{c}}_{i}^{mol}V_{w}^{free}/V_{tot},$$

where $V_{w}^{free}$ is the volume of free water in one equivalent of the membrane.

Since according to our calculations, $V_{w}^{free}/V_{tot}$ ≈ 1/3, *K_D_* in Equation (12) should be of the order of 0.1.

The volume fraction of macropores in the MA-41 membrane is estimated as *f*_macro_ = 0.1. This quantity was determined by Vasil’eva et al. [66,67] by digital processing of SEM images. This result agrees with the evaluation of *f*_macro_ by determining the volume fraction of water filling the membrane pores with the radius from 100 to 1000 nm [55], when using the pore distribution diagrams reported by Kononenko et al. [68]. PPy is a polymer that has relatively large pores [38,52]. When swelling in an aqueous solution, the pore size of PPy should increase. Therefore, the parameter *f*_macro_ for PPy must be significantly larger than that for a dense MA-41 membrane; we set *f*_macro_ = 0.83 for the freshly synthesized wet PPy, and *f*_macro_ = 0.40 for the wet PPy in the state after the use of the membrane in ED (PPy_ED_).

The hydration numbers of Cl^−^ and Na^+^ and that for the fixed quaternary amine ion were taken from [55], where they were estimated using the literature data on a swelling experiment of an ion-exchange resin [1], on an ion mobility in solution [69], on the partial molar volumes of NaCl, NaBPh_4_, and Ph_4_AsCl in water solutions [70] and on the membrane water content [66].

The structural parameter $\alpha^{\prime }$ and the character diffusion coefficients ${\bar{D}}_{i}^{\prime }$ of mobile ions in the gel phases of the host membrane and nested PPy are fitting parameters. In principle, different sets of the input parameters make it possible to satisfactorily describe the experimental data (membrane conductivity, diffusion permeability and thickness) considered in the next Section. However, there is a certain logic in the accepted values presented in Table 1. The value of $\alpha^{\prime }$ in the MA-41 membrane is set equal to 0.15, which is typical for heterogeneous membranes [40,56]. The MA-41_mod_ membrane has a stretched matrix, which should provide easier pathways for ions, since some very narrow pores become larger and ions do not need to go around them (Figure 5). Stretching of the matrix leads to a reduction in series connections of the gel and intergel solution and an increase in parallel connections. For this reason, we set $\alpha^{\prime }$ = 0.42 for MA-41_mod_ and 0.23 for MA-41_modED_. The latter is due to the fact that after ED the membrane thickness decreased, the degree of stretching was reduced, but the membrane thickness remained higher than that of the pristine membrane. Therefore, the value of $\alpha^{\prime }$ for the MA-41_modED_ membrane is between the values of these parameters for MA-41 and MA-41_mod_. As for the nested PPy, $\alpha^{\prime }$ was set −0.28 for the freshly prepared membrane and 0.08 for that used in ED. The first value is explained by the specific structure of this polyelectrolyte: there are relatively large free spaces limited by polymer chains wearing charges with a high local density (5.1 mmol/cm^3^ dry PPy [50]) (Figure 5). Although counterions can easily pass through the charged walls, coions have to find gaps in the polyelectrolyte chains where the local concentration of the fixed charges is low in order to pass from one solution-filled space to the next one. The pathway of coions is quite tortuous and is characterized by a great number of series connections. The concentration of fixed charges in the chains of PPy that underwent an ED operation together with the host membrane is much lower; hence, coions easily find pathways to move from one solution-filled space to the next. This makes the value of $\alpha^{\prime }$ larger and closer to that of the pristine membrane.

Similar logic was used when choosing the $D_{i}^{\prime }$ values. The counterion diffusion coefficient, ${\bar{D}}_{Cl}$, in the gel phase of MA-41_mod_ is greater than that in MA-41 because of the stretching of the matrix. However, ${\bar{D}}_{Na}$ is lower in the gel, since coions have to pass around the mesopores filled with PPy, while in MA-41 they pass relatively easily through these regions. For a similar reason, ${\bar{D}}_{Cl}$ in the freshly prepared PPy is quite high, while ${\bar{D}}_{Na}$ is very low. After the use in ED, ${\bar{D}}_{Cl}$ in PPy decreases, since the loss of fixed charges leads to a loss of water and narrowing the pores permeable to ions. In the contrary, ${\bar{D}}_{Na}$ increases, as the biggest barriers for coions are fixed charges of the same sign. However, this coefficient remains lower than ${\bar{D}}_{Cl}$.

Young’s modulus is found by fitting the values of the membrane thickness measured for the pristine and modified membranes (Section 3.2). The value of this modulus for the modified membranes is nearly two times lower than for the pristine membrane. This result reflects a decrease in the membrane mechanical strength caused by its modification. The mechanical strength, i.e., the capability of the membrane to resist stretching caused by internal osmotic pressure, depends on the ability of the membrane’s polymer chains to stretch elastically. After modification, the membrane matrix is significantly stretched due to the increased osmotic pressure created by additional fixed charges. Apparently, part of the polymer chain breaks, which is the reason for the decrease in the Young modulus.

### 3.2. Results of Simulations

**Figure 6:**
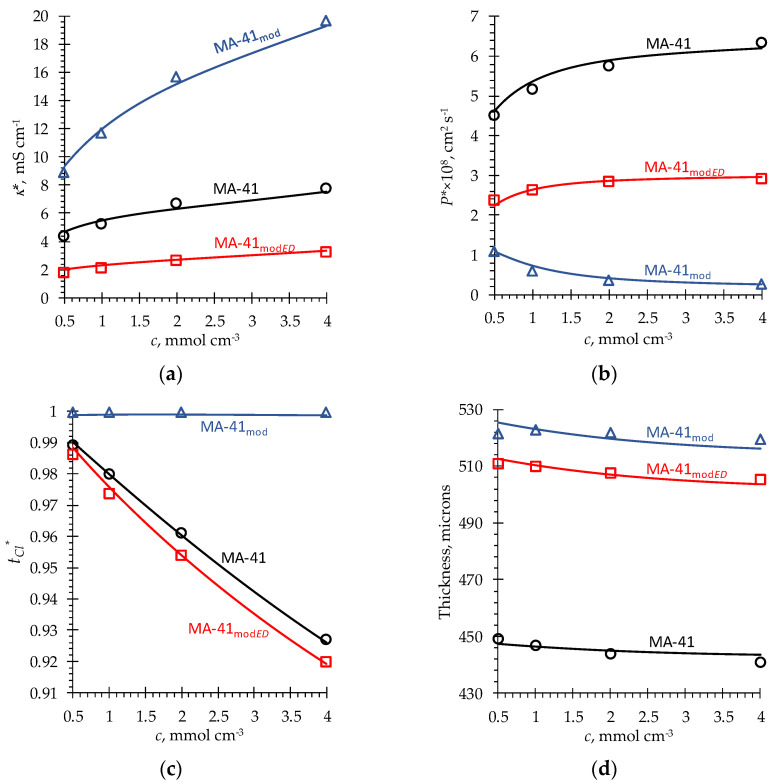
Experimental (markers) and simulated (solid lines) concentration dependencies of (**a**) electric conductivity, (**b**) diffusion permeability, (**c**) counterion, Cl^−^, transport number and (**d**) thickness of MA-41, MA-41_mod_ and MA-41_mod*ED*_ membranes (indicated near the corresponding curve).

As can be seen from Figure 6, the choice of input parameters (Table 1) described in the previous Section makes it possible to obtain a quantitative agreement between the experimental and simulation results for the MA-41, MA-41_mod_ and MA-41_mod*ED*_. A good agreement is obtained at once for electric conductivity, diffusion permeability, transport numbers of Cl^−^ ions and membrane thickness as functions of the concentration of bathing solution.

As noted above, some input parameters ($\alpha^{\prime }$, ${\bar{D}}_{i}^{\prime }$) and some output parameters (κ*, P*, *t*_Cl_*, thickness, *q_wb_*, *q_w_*, *f*_2*m*eso_) are functions of the bathing solution concentration *c*. These functions are presented in Figure S1, Supplementary Material. Table 1 gives the characteristic values of $\alpha^{\prime }$ and ${\bar{D}}_{i}^{\prime }$. The concentration dependencies of the main membrane characteristics are shown in Figure 6; in order to facilitate the comparison of some output parameters in the pristine membrane and in the different states of its modification, the values of these parameters at *c* = 1 M NaCl are gathered in Table 2.

Figure 6 shows that the conductivity of the MA-41_mod_ membrane and the Cl^−^ transport number in this membrane are significantly higher than these characteristics for the pristine membrane. With that, the diffusion permeability is much lower. However, after the use of this membrane in an ED process at current densities close to or higher than the limiting over 5 h, the values of κ* and *t*_Cl_* become lower than those for the pristine MA-41 membrane. The value of P* increases, but remains lower than that for the MA-41 membrane. The cause of these changes in the membrane properties during ED is the degradation of PPy due to water splitting (Section 3.1). Note that an increase in the conductivity of an AEM after its PPy modification was also experimentally detected by Salmeron-Sanchez et al. [38]. The authors also gave a theoretical explanation for this effect on the basis of the MHM, assuming that PPy bearing anion-exchange functional groups occupies the pores of the host membrane. This increases the concentration of counterions in the gel phase of the membranes, which leads to an increase in membrane conductivity. Our model also supports this explanation. In addition, it is important that the membrane stretches after the PPy modification. This stretching leads to an increase in the value of ${\bar{D}}_{Cl}$ and the value of electroneutral solution filling the central parts of macro- and meso-pores, *f*_2_, in the freshly modified membrane. The value of *f*_2_ increases in spite of the fact that PPy occupies the macro- and mesopores. The reason is that PPy is a polyelectrolyte, which has relatively large pores in the swollen state [52]. The model assumes that 83% of the space occupied by PPy remains macropore. These macropores make a significant contribution to the high membrane conductivity, especially in concentrated solution, whose specific conductance is much higher than that of the gel phase at *c* > 1 mol/L.

At first glance, it seems that a very high conductivity of the MA-41_mod_ membrane contradicts the fact that its diffusion permeability is very low (Figure 6). However, the model explains this effect by a very low effective mobility of coions in the macropores occupied by PPy. As mentioned in the previous Section, a high concentration of positive fixed charges in the polymer chains forming walls between solution-filled spaces presents a big barrier for coions. This feature of the PPy structure is taken into account in the model by a very low coion diffusion coefficient in PPy and a negative value of *α* in the spaces occupied by PPy. Negative values of *α* reflect the fact that series connections of the gel and intergel solution predominate over parallel connections (Section 3.1). Since both the gel phase and the intergel solution are good conductors for counterions, the value of *α* has a relatively weak effect on the membrane conductivity. However, the gel phase is difficult to overcome for coions. For this reason, decreasing *α*, especially when it is negative, strongly reduces salt diffusion.

Note the unusual concentration dependence of P* in the MA-41_mod_ membrane: instead of an increase in P* with an increase in *c*, generally detected in experiments [40,46,71], we see a decreasing concentration dependence. Usually, an increasing P* vs. *c* curve is explained by the fact that the coion concentration in the membrane increases with an increase in *c* due to weakening of the Donnan exclusion effect. In the case of MA-41_mod_, the Donnan exclusion weakens with an increase in *c*. However, the effect of a decrease in the parameter *α* with an increase in the bathing solution concentration and the fact that *α* < 0 are of greater importance. This effect is caused by a decrease in the membrane pore size due to the loss of water by the membrane in a concentrated solution and an increase in the tortuosity of the coion pathway, as discussed in Section 3.1. As seen in Figure S1 (Supplementary Materials), *α* decreases with increasing *c* in all cases: for the host membranes (MA-41, MA-41_mod_ and MA-41_modED_), and for PPy in these membranes. However, only in the case of the freshly prepared PPy is *α* < 0. In the case of PPy in the membrane used in ED, *α* is small, but positive. This difference in *α* between the “fresh” PPy and PPy in the used membrane is explained by the loss of fixed charges on the PPy chains caused by the attack of OH^−^ ions during ED, as described in Section 2.1.4. A decrease in the exchange capacity of PPy facilitates coion transport through the PPy chains: coions do not need to find long and tortuous pathways to transfer through a region occupied by PPy. As a consequence, the fraction of the series connections decreases, and *α* increases.

The fraction of mesopores occupied by the electroneutral solution, *f*_2meso_, calculated using Equations (20)–(23) is equal to 0.1 for the pristine MA-41, as in Ref. [55]. This value is characteristic for homogeneous ion-exchange membranes, not containing macropores, when it is understood as the volume fraction of electroneutral intergel solution in these membranes [55]. The model shows that *f*_2meso_ is slightly higher in the MA-41_mod_ (*f*_2meso_ = 0.12) and MA-41_modED_ membranes (*f*_2meso_ = 0.14) (Table 2), since the matrix of the MA-41 membrane is stretched, which is characterized by a higher size of all pores. The obtained values are consistent with the values of the bound and free water (Table 2) involved in Equations (20)–(23). When a mesopore increases in size, the EDL thickness does not change, therefore the fraction of the electroneutral solution in the pore increases. In addition, some micropores may become mesopores after stretching the matrix.

The modelling allows us to formulate the main properties of a modifier capable of imparting such high characteristics to the host ion-exchange membrane. The modifier should (1) be a polyelectrolyte bearing fixed functional groups with the same sign of charge as that the host membrane; (2) be nested into the non-selective macro- and mesopores of the host membrane; (3)have a special structure: the polymer chains should form charged walls that separate the solution in a macropore into smaller compartments. The ions in the solution filling these compartments should be sufficiently mobile to ensure high conductivity of the membrane in concentrated external solutions.

## 4. Conclusions

We show that a PPy-based modification of a heterogeneous AEM with quaternary ammonium functional groups can lead to a membrane of exceptional performance. Its conductivity reaches almost 20 mS/cm and the chloride transport number *t*_Cl_* > 0.99 in 4 M NaCl. The modelling carried out in this work serves to understand what parameters an IEM may have to show such exclusive parameters, especially a very high counterion permselectivity.

Although PPy as a modifier makes it possible to obtain a membrane with extraordinary performance (when the membrane with PPy is freshly prepared), this polymer is not stable. It degrades, in particular, under attacks of OH^−^ ions, which can be a product of water splitting at the membrane/solution interface. Therefore, we cannot give a positive answer to the question posed in the title, at least if the same membrane modification protocol is used as described in this paper. However, our results can tell what properties the modifier polymer should have to preparer a “super” membrane. Another polyelectrolyte with similar properties but stable, or a method of PPy stabilization, are needed to obtain better membranes.

## Figures and Tables

**Table 1:** The values of the input parameters of the model.

Parameter	MA-41	MA-41_mod_	MA-41_modED_	PPy	PPy_ED_	Description
Thermodynamic parameters						
*Q*, mmol/cm^3^ swollen membrane	1.47	1.47	1.47	2.0	0.05	Membrane exchange capacity
*K_D_*	0.035	0.09	0.05	0.01	0.10	Donnan equilibrium constant
Structural parameters						
*f*_macro_	0.10	0.10	0.10	0.83	0.40	Volume fraction of macropores
$\alpha^{\prime }$	0.15	0.42	0.23	−0.28	0.08	Parameter of the membrane structure
$V_{Rdry}$, cm^3^/mmol	0.48	0.48	0.48	0.03	0.03	Volume of dry polyelectrolyte gel containing 1 mmol of fixed functional groups with counterions
*h_R_*	1.7					Hydration number of membrane fixed groups
*h_Cl_*	0.5					Hydration number of Cl^−^ in the membrane pore solution
*h_Na_*	1.5					Hydration number of Na^+^ in the membrane pore solution
Kinetic parameters						
$D_{Cl}^{\prime }$, cm^2^/s	3.4 × 10^−7^	5.8 × 10^−7^	2.0 × 10^−7^	5.4 × 10^−7^	1.1 × 10^−7^	The values of Cl^−^ and Na^+^ diffusion coefficients in the gel phase at *c* = 1 M NaCl, Equation (24)
$D_{Na}^{\prime }$, cm^2^/s	6.0 × 10^−8^	3.5 × 10^−9^	3.2 × 10^−8^	6.4 × 10^−10^	4.6 × 10^−8^	
Mechanical parameter						
*E*, bar	3800	2000	2000	-	-	Young’s modulus

**Table 2:** The values of some output parameters of the model at *c* = 1 M NaCl.

Parameter	MA-41	MA-41_mod_	MA-41_modED_	PPy	PPy_ED_	Description
*f*_2*m*eso_	0.1	0.12	0.14	0.12	0.14	The volume fraction of the electroneutral solution localized in the membrane mesopores, Equation (20)
*f*_2_	0.2	0.24	0.24	0.95	0.55	The volume fraction of the intergel spaces, Equation (19)
*q_wb_*	2.2	3.0	2.4	-	-	The amount of bound water in the membrane
*q_w_*	6.0	9.7	9.0	-	-	The amount of free water in the membrane
*ε*	0.088	0.120	0.118	-	-	Relative elongation, Equation (10)

## Supplementary Materials

The following supporting information can be downloaded at: https://www.mdpi.com/article/10.3390/membranes13010103/s1, Figure S1: Simulated concentration dependencies of (a) tortuosity coefficient (Equation (25)), (b) *f*_2_ (Equation (19), solid lines) and *f*_2meso_ (Equation (20), dashed lines), (c) counterion, Cl^−^ (solid lines), and coion, Na^+^ (dashed lines), diffusion coefficients in membrane gel phase (Equation (24)), (d) parameter α (Equation (27)), (e) *q_wb_* (Equations (6) and (32), solid lines) and *q_w_* (dashed lines), (f) membrane water content. Dependencies for MA-41, MA-41_mod_ and MA-41_mod*ED*_ membranes as well as PPy or PPy_ED_ are indicated near the corresponding curve. References [1,52,72] is cited in the Supplementary Materials.
