# Supplementary material for: Is It Possible to Prepare a “Super” Anion-Exchange Membrane by a Polypyrrole-Based Modification?
Source: Membranes (Basel). 2023 Jan 12;13(1):103. doi: 10.3390/membranes13010103 (PMC9865286; doi:10.3390/membranes13010103)
Supplement: Supplementary file 1 [file membranes-13-00103-s001.zip › membranes-2152282-supplementary.pdf]

Supplementary Materials

Is It Possible to Prepare a "Super" Anion-Exchange Membrane by a Polypyrrole-Based Modification?

Anton Kozmai, Mikhail Porozhnyy, Valentina Ruleva, Andrey Gorobchenko, Natalia Pismenskaya and Victor Nikonenko *

Membrane Institute, Kuban State University, 149, Stavropolskaya Str., 350040 Krasnodar, Russia

* Correspondence: v_nikonenko@mail.ru; Tel.: +7-918-41-45-816

Some output parameters as functions of the bathing solution concentration.

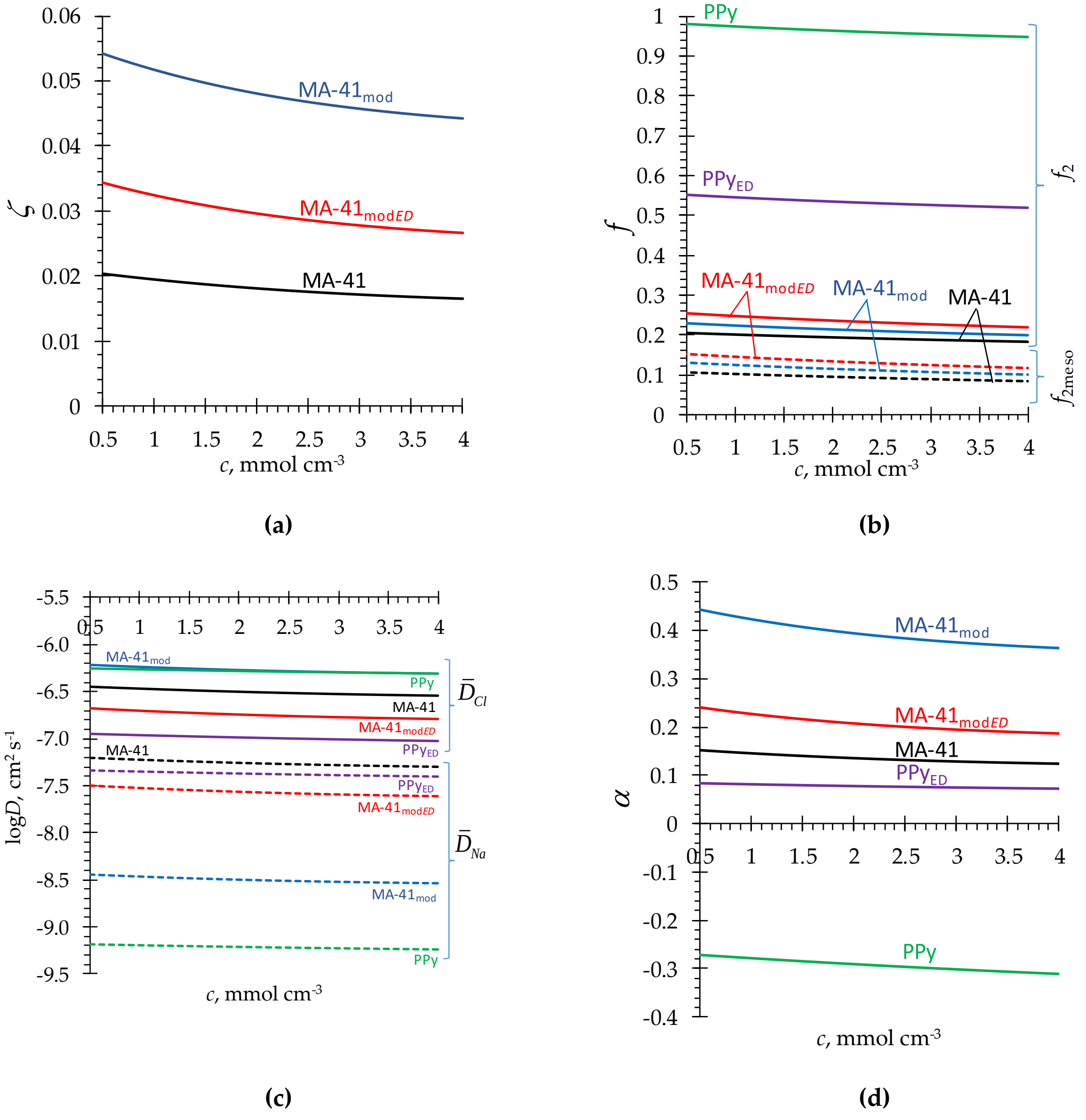

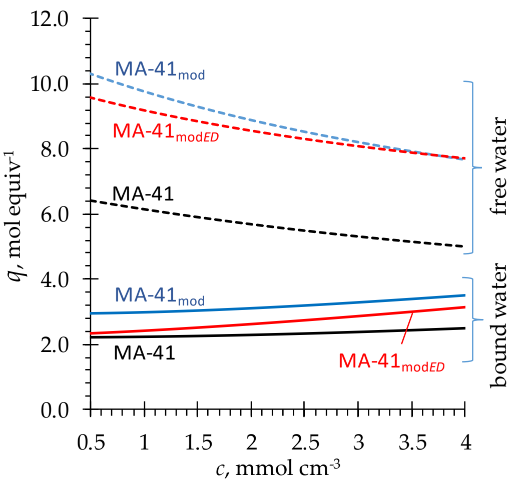

(e)

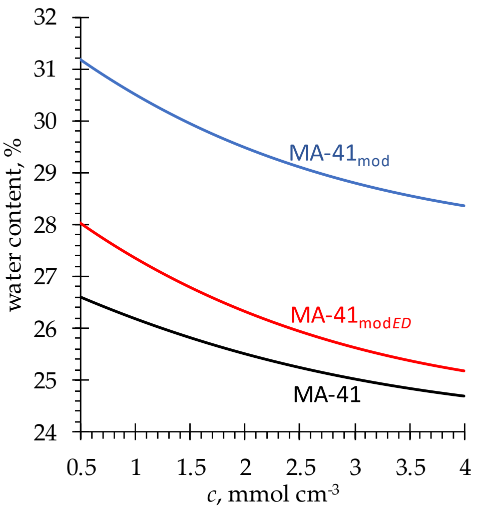

(f)

Figure S1. Simulated concentration dependencies of (**a**) tortuosity coefficient (Eq. (25)), (**b**) f_2 (Eq. (19), solid lines) and f_{2meso} (Eq. (20), dashed lines), (**c**) counterion, Cl^- (solid lines), and coion, Na^+ (dashed lines), diffusion coefficients in membrane gel phase (Eq. (24)), (**d**) parameter α (Eq. (27)), (**e**) q_{wb} (Eqs. (6) and (32), solid lines) and q_w (dashed lines), (**f**) membrane water content. Belonging of dependencies to MA-41, MA-41$_{mod}$, MA-41$_{modED}$ membranes, PPy or PPy$_{ED}$ is indicated near the corresponding curve.

Note that in Figure S1b, the f_{2meso} values for the host membrane and the corresponding nested polypyrrole are the same.

Evaluation of V_{Rdry} for the host matrices of the studied membranes and for PPy.

V_{Rdry} for the host matrix of MA-41 was found from the definition of the equivalent volume, which leads to the relation [1,72]: $V_{Rdry}^{MA41} = 1/\bar{Q}_{dry}^{MA41} = (1 - f_{macro})/Q_{dry}^{MA41}$; where the exchange capacity of the dry pristine membrane, Q_{dry}^{MA41}, found experimentally is equal to 1.89 mmol/(cm^3 dry MA-41 membrane); $\bar{Q}_{dry}^{MA41}$ is the exchange capacity of the dry polyelectrolyte gel not containing macropores (in mmol/(cm^3 dry gel). We accept f_{macro} = 0.1, then $\bar{Q}_{dry}^{MA41}$ = 2.1 mmol/(cm^3 dry gel). In the model, we suppose that V_{Rdry} of the host membrane matrix does not change (Table 1). Thus, the value of V_{Rdry} for the modified membrane is taken the same for MA-41$_{mod}$ and MA-41$_{modED}$ as for the pristine membrane.

If we accept the assumptions presented in Section 3.1 concerning PPy, we can evaluate the volume of the dry PPy in the equivalent volume of the MA-41$_{mod}$ membrane. It is supposed that PPy fills the intergel spaces (i.e. macropores and central parts of the mesopores occupied by electroneutral solution in the pristine membrane) (Figure 5b). Taking into account that the volume fraction of the intergel spaces in the membrane is f_2, we find

$$V_{Rdry}^{PPy} = \frac{V_{Rwet}^{\mathrm{mod}} f_2 (1 - f_{macro}^{PPy}) f_{PPy}^{w}}{(1 - f_{macro}^{\mathrm{mod}})}, \qquad \text{(S1)}$$

where f_{PPy}^{w} is the ratio of the volume of PPy to the volume of water in the parts of this polymer not containing macropores.

However, the spaces occupied with PPy also contain macropores due to a specific volumetric structure of this polymer [52]. If we set the volume fraction of the macropores in the spaces occupied with PPy, f_{macro}^{PPy}, equal to 0.83 (Table 1), $f_{PPy}^{w} = 0.8$, f_{macro}^{mod} = 0.1 (Table 1), we find $V_{Rdry}^{PPy} = 0.03$ (Table 1).
